# EGNet: 3D Semantic Segmentation Through Point–Voxel–Mesh Data for Euclidean–Geodesic Feature Fusion

**DOI:** 10.3390/s24248196

**Published:** 2024-12-22

**Authors:** Qi Li, Yu Song, Xiaoqian Jin, Yan Wu, Hang Zhang, Di Zhao

**Affiliations:** 1 School of Computer Science and Technology, Changchun University of Science and Technology, Changchun 130022, China; 2 Jilin Provincial International Joint Research Center of Brain Informatics and Intelligence Science, Changchun 130022, China; 3 Zhongshan Institute of Changchun University of Science and Technology, Zhongshan 528437, China

**Keywords:** geodesic information, neural network, point cloud, semantic segmentation, voxel data

## Abstract

With the advancement of service robot technology, the demand for higher boundary precision in indoor semantic segmentation has increased. Traditional methods of extracting Euclidean features using point cloud and voxel data often neglect geodesic information, reducing boundary accuracy for adjacent objects and consuming significant computational resources. This study proposes a novel network, the Euclidean–geodesic network (EGNet), which uses point cloud–voxel–mesh data to characterize detail, contour, and geodesic features, respectively. The EGNet performs feature fusion through Euclidean and geodesic branches. In the Euclidean branch, the features extracted from point cloud data compensate for the detail features lost by voxel data. In the geodesic branch, geodesic features from mesh data are extracted using inter-domain fusion and aggregation modules. These geodesic features are then combined with contextual features from the Euclidean branch, and the simplified trajectory map of the grid is used for up-sampling to produce the final semantic segmentation results. The Scannet and Matterport datasets were used to demonstrate the effectiveness of the EGNet through visual comparisons with other models. The results demonstrate the effectiveness of integrating Euclidean and geodesic features for improved semantic segmentation. This approach can inspire further research combining these feature types for enhanced segmentation accuracy.

## 1. Introduction

As home service robot technology advances, the demand for three-dimensional (3D) indoor scene understanding has increased. While an outstanding level of performance in robotic object grasp detection has been achieved, it is essential to identify the spatial layout of the interior environment, the type of object, and the positional relationship between objects [1,2]. Therefore, 3D scene understanding for indoor environments has become increasingly significant, with this study focusing on the 3D semantic segmentation of indoor scenes.

**Figure 1:**
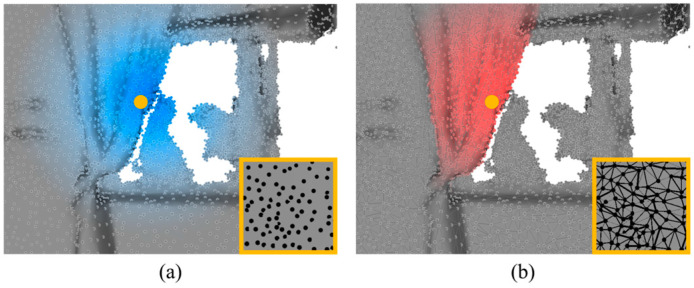
The yellow point on the curtain serves as the focal point for all color shades that represent the distance within the neighborhood. The shades of color represent the Euclidean distance between each point and the focal point in point cloud data (blue), shown in (**a**). The shades of color represent the path length between each point and the focal point (red) in 3D mesh data, shown in (**b**). The data structures of each are displayed in the yellow bounding boxes.

Three-dimensional point cloud semantic segmentation assigns semantic labels to individual points, linking each point to a label that represents a real category. However, point cloud data are characterized by their disordered distribution, uneven density, and sparsity, which makes it challenging to effectively apply traditional convolutional methods. Consequently, scholars have proposed voxelization, a method that transforms point clouds into dense, normalized data, enabling the use of a standard 3D convolution for segmentation [3,4]. Nevertheless, the methods often result in the loss of intricate shape details while retaining the sparsity of point clouds. Liu et al. [5] proposed supplementing point cloud features with voxel data and incorporating multimodal data to improve local refinement. By using Euclidean convolution, both point cloud and voxel data can bridge small gaps, facilitating the propagation and accumulation of neighborhood information between spatially adjacent objects. As shown in Figure 1a, objects such as windows and walls are typically detected beside a curtain by learning the Euclidean local point neighborhood. Although these methods have shown some results in Euclidean domains, constructing topological structures of point sets solely on the Euclidean distance between points does not explore surface information. Because various objects are spatially close to each other, semantic segmentation models may mistakenly recognize unconnected objects as connected within local neighborhoods. The mesh data structure approximates an object’s surface by a set of two-dimensional polygons in 3D space. The boundary of the complex object can be separated in a mesh, distinguish between easily confused front–back topologies, and provide the intrinsic connection and information representation of the point set. By utilizing mesh data, curtains can be better distinguished from other objects using mesh data, as shown in Figure 1b. By capturing information regarding the mesh path length to the object’s surface topology, even physically similar objects can be accurately distinguished by the difference in the length of their surface paths, enabling efficient boundary segmentation.

This study proposes a Euclidean–geodesic network (EGNet) that contains Euclidean and geodesic branches. The Euclidean branch extracts the detailed features and voxelated contour features of point cloud data. The geodesic branch extracts geodesic features from mesh data using graph message propagation methods and fuses these with contextual features extracted from the Euclidean branch for point cloud semantic segmentation. The primary challenge addressed in this study is the process of effectively combining the features of point, voxel, and mesh data. Consequently, we proposed a self-attention module and a cross-domain attention module based on the feature fusion method from reference [6]. The self-attention module uses the unified message passing model to focus its attention on the vertex features of mesh data. The cross-domain attention module uses the same model to achieve attention fusion. The feature of the Euclidean branch is fused with the vertex feature of the mesh data to achieve the integration of point cloud, voxel, and mesh data. The EGNet is evaluated using two large-scale real-world datasets, Scannet and Matterport, and achieved satisfactory performance. Notably, the accuracy of some results even surpassed that of the manually annotated labels.

## 2. Related Work

### 2.1. Method Based on Euclidean Feature

Many researchers [7,8,9,10] have proposed point-based approaches. However, a significant portion of the processing time, approximately 90%, is spent on converting irregular data into structured neighborhood representations rather than on actual feature extraction. PtV2 [11] uses a fractal geometry approach to record the mapping generated through serialization to transform unstructured and irregular point clouds into regular and serialized data. While this method yields better results, it leads to the loss of boundary information. Another method divides the 3D space into regular voxels before the regular convolution of the voxels [12]. Although these approaches improve the efficiency of data access, voxelization incurs a loss of information and high computational and memory overheads when handling large-scale scenarios. To further improve the efficiency of 3D deep learning, Liu et al. proposed a method based on point–voxel convolution [5]. This method consists of two parts: the point-based branch, which processes each point directly, and the voxel-based branch, which fuses the voxelization results of the point-based branch before performing the convolution. Although it performs well for understanding small regions, it is not applicable to large-scale scenes. Therefore, a sparse convolution (SC) [13] is applied to the point–voxel fusion network to address the complexities of voxel calculations.

### 2.2. Method Based on Geodesic Feature

The geodesic distance measures the shortest path between any two vertices, calculated in two ways: the first method uses the inherent shape information of the object’s surface [14,15] and the second provides an approximate solution by constraining the shortest length of the path on graph data [16,17]. Both methods utilize the surface information of mesh data to construct graph data structures and extract features from these structures through a graph convolution. This approach also influenced our design philosophy and motivated the development of the EGNet. We perform element-wise multiplication between the weight matrix in the attention mechanism and the feature matrix constructed from the graph data, incorporating regularization to extract the relevance between two connected points. If the points belong to different labels, the corresponding elements in the weight matrix should approach zero.

### 2.3. Method Based on Geodesic Features and Euclidean Features

To simultaneously extract the features from point cloud and mesh data, many scholars have proposed solutions that fuse geodesic and Euclidean features. The DualConvMesh-Net model [18] uses a radius graph to define the concept of neighborhoods for vertices in the Euclidean space. Euclidean and geodesic features are extracted by using graph convolutions and dynamic graph convolutions, respectively, and subsequently, the resulting feature map is concatenated. The DualConvMesh-Net combines the benefits of 3D surface meshes and a Euclidean graph convolution on 3D vertices in the spatial domain. The VMNet [19] uses voxelization and SC to extract the Euclidean spatial features in the Euclidean module, and the voxel features are projected onto the mesh features by a linear interpolation for further geodesic convolution. In our method, a multi-layer perceptron (MLP) is applied to each point data to extract high-frequency features, compensating for the information loss caused by voxelization.

## 3. Methodology

**Figure 2:**
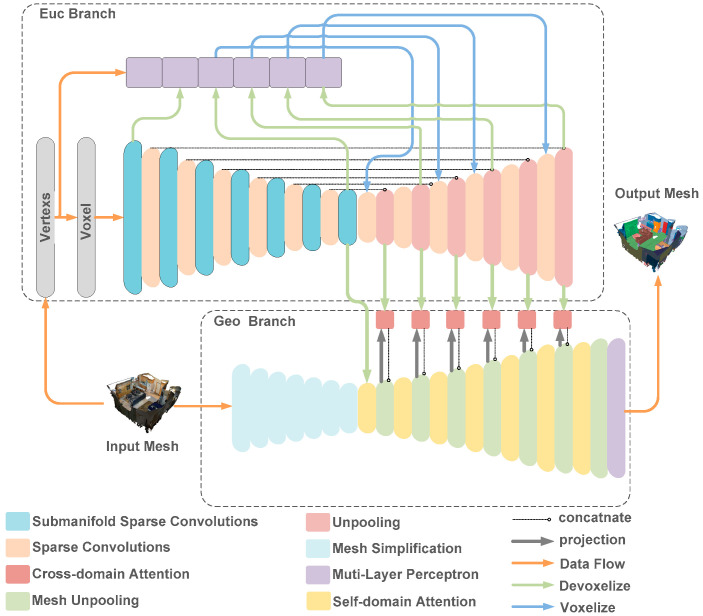
EGNet architecture. In the Euclidean branch, we use a feature extractor similar to U-Net structure for extracting Euclidean features from voxels to capture fine features. Inspired by PointNet++ structure, we incorporate a point-based MLP into the Euclidean branch. In the geodesic branch, the self-domain attention module is used to effectively aggregate the vertices of the original mesh. The features of the mesh vertices are fused with the features of sparse vertices from Euclidean branch using the cross-domain attention module.

We proposed an indoor point cloud semantic segmentation network, named EGNet. EGNet processes three types of 3D representations: points, voxels, and meshes. As depicted in Figure 2, the network consists of two branches: the upper one is denoted as the Euclidean branch and the lower as the geodesic branch.

In the Euclidean branch, we use a feature extractor similar to the U-Net structure to extract Euclidean features from voxels to capture fine features. Additionally, inspired by the PointNet++ structure, we incorporate a point-based multilayer perceptron (MLP) to extract features from individual points and capture high-frequency features (i.e., fine details). However, the dense sampling of point cloud data in certain areas can lead to an over-reliance on the weight of the calculation of point cloud data in dense areas. Consequently, we fuse the SC and the submanifold convolution [20] to extract low-frequency features, while the MLP compensates for high-frequency features. Specifically, MLP focuses on extracting high-frequency features, and SC and submanifold convolution are used to extract low-frequency features. Sparse deconvolution is applied to aggregate high-frequency and low-frequency features, enhancing the model’s segmentation efficiency. In the geodesic branch, vertex clustering (VC) and quadratic error metrics are used to simplify the mesh, which represents non-Euclidean data. This simplification records trajectory maps for the upsampling process. Subsequently, the mesh is transformed into a graph structure, in which a cross-domain attention module is used to aggregate the features extracted from the Euclidean branch with those from the self-domain attention module. Finally, the aggregated features undergo processing through the self-domain attention module and are upsampled further. Inspired by reference [19], this process is repeated six times to achieve satisfactory performance. A visual representation of the data processing flow of EGNet is depicted in Figure 2. While in the geodesic branch, a self-domain attention module is used to efficiently aggregate the vertices of the original mesh, the features of the mesh vertices are fused with the sparse vertex features from the Euclidean branch via a cross-domain attention module.

### 3.1. Euclidean Branch

(1) MLP based on point: Sparse voxel branches cannot model fine single-point features effectively. Inspired by the work of H. Tang [9], we improve the method for combining point features and voxel features of point cloud data. The MLP is used to extract the features of a single point and achieve high-resolution point information to compensate for the missing information based on coarse-grained voxels. We employ voxelization and devoxelization to achieve data consolidation during fusion.
(2) Voxelization: The point cloud tensor is expressed as *C* = ({*P_c_*, *F_c_*}), where *P_c_* = (*x_i_*, *y_i_*, *z_i_*) defines the 3D coordinates of the point cloud and *F_c_* represents the feature vector of the points [21]. The sparse tensor is described as *S* = ({*P_s_*, *F_s_*}, *v*), where *P_s_* is the ratio of the 3D coordinates of the point cloud to the voxel size, *F_s_* is the feature tensor of the grid, and *v* is the voxel resolution.

In the voxel branch, we rasterize the point cloud and convert the point cloud tensor C into sparse tensor I as the network input. Equations (1) and (2) describe the function of voxelization.

(1) $$P_{s}=\left(\tilde{x_{i}},\;\tilde{y_{i}},\;\tilde{z_{i}}\right)=\left(floor\left(\frac{x_{i}}{v}\right),\;floor\left(\frac{y_{i}}{v}\right),\;floor\left(\frac{z_{i}}{v}\right)\right)$$

(2) $$F_{s}=\frac{1}{N_{m}}\sum_{k=1}^{n}1\left[P_{s}\right]\cdot F_{c}$$

where 1[·] is a binary indicator indicating whether *P_c_* is included in the grid. *N_m_* is the normalization coefficient representing the number of points located in the *m*-th non-zero voxel, and the floor function rounds down.

(3) Devoxelization: In the devoxelization process, inspired by [17], we use trilinear interpolation at each layer to transform the eight neighboring voxel rasters into a point cloud tensor. The features of each point are interpolated using the features of the eight neighboring voxels. Note that, for the de-voxelization process, if we use the voxelized data converted to point cloud data, these point cloud data will generate a rough 3D mesh, causing information loss. To avoid this, one option is to increase the voxel mesh resolution; however, this method will cause memory overflow. Therefore, we use the original 3D point cloud as nodes based on the approach for model inference used in [17]. For each point $O_{i}=\left(P_{i}^{x},\;P_{i}^{y},\;P_{i}^{z}\right)$, we define a random variable $x_{i}$ to denote the relationship between data categories (semantic information). We also define $L_{i}\left(x_{i}\right)$ to be the scores (logits) associated with the probability distribution of $x_{i}$, where $L_{i}$ is operated as shown in Equation (3):

(3) $$L_{i}\left(x_{i}\right)=\sum_{n=1}^{8}w_{i,n}L_{i,n}\left(x_{i,n}\right)$$

That is, $L_{i}$ is the weighted sum of the scores of the eight spatially nearest neighbor voxels $V_{i,n}\;\left(n\in \{1,\ldots ,8\}\right)$.

Our approach differs from that of [17] with respect to the operation procedure of $w_{i,n}$, considering that the goal of this work in the literature [17] is to implement the gradient feedback of conditional random fields (CRFs) in backpropagation by using $w_{i,n}$. In the original literature, the word “splat” is used to define the process of “feedback” 3D-FCNN by obtaining the score to be used as a monadic term in the CRFs.

Therefore, the procedure for $w_{i,n}$ in [17] is $w_{i,n}=\Pi_{s\in \{x,y,z\}}\left(1-\left|P_{i}^{s}-P_{i,n}\right|/V\right)$, where *V* denotes the size of the voxel. We compute $w_{i,n}$ as shown in Equation (4):

(4) $$w_{i,n}=\frac{1}{S_{xyz}}\cdot \frac{1}{{∥P_{i}-P_{i,n}∥}_{V}}$$

where $S_{xyz}$ is the size of the voxel and ${∥\cdot ∥}_{V}$ denotes the distance in voxel space. We use the inverse of the distance as a weight, implying that a voxel center $P_{i,n}$ that is closer to the target point $P_{i}$ will have a greater impact on the category score of the target point. This is because voxel centers closer to the target point are more representative of the features or categories of the target point. This operation will increase the difference in the weight vectors corresponding to different categories of points positioned at the edge, which is more conducive to the segmentation of the boundary.

(4) Combination of sparse convolution and submanifold sparse convolution: Generally, the sparse tensors are convolved in a certain access order. However, the conventional convolution operation is not adjusted to suit the sparsity of the input data, resulting in the “submanifold dilation problem”. Submanifold sparse convolution (SSC) [20] addresses the limitation of submanifold dilation by restricting the convolutional output to a group of active input regions to ensure the sparsity stays constant across multiple layers. However, SSC causes each pixel to be processed separately, which limits the network’s ability to extract the correlation with neighborhood information. Therefore, our method combines the SC (*m*, *n*, *f*, *s* = 2) and SSC (*m*, *n*, *f*, *s* = 1) to create convolution networks based on U-Net, where *m* is the input features, *n* is the output features, *f* is the filter size, and *s* is the step size. The size of the output feature map is (*l* − *f* + *s*)/*s*.

Given that the method [22] can only perform matrix multiplication on a single kernel, we construct the hash table and a rulebook for activating input sites in the sparse tensor I inspired by the TorchSparse [23] to achieve parallel computation on the GPU. The hash table contains the index and position tuples of all activated input sites, while the rulebook records the input positions of the activation sites and the output positions generated by convolution with the kernel. SC and SSC are used to perform neighborhood feature aggregation for sparse tensors. We parallelize kernel mapping operations on the GPU based on the created hash table. The voxelized coordinate of each point in the point tensor Z is used to search for the index in the sparse tensor, which improves the search efficiency over sequential search.

### 3.2. Geodesic Branch

(1) MLP based on point cloud data: Mesh simplification primarily minimizes the number of triangular meshes while preserving the model’s geometric details and texture components. Developed methods for mesh simplification include VC [24], edge collapse quadratic error metric (QEM) [25], wavelet discretization [26], and a combination of VC and QEM [19]. Our experiments compare these methods, revealing that edge collapse yields optimal performance. Although the literature [19] shows that direct application of the QEM method produces high-frequency noise signals and that combining clustering and edge folding methods is optimal, we have already extracted the high-frequency features from the point cloud data using the MLP network in the Euclidean branching. Therefore, we do not need the high-resolution features embedded in the gridded data prior to simplification. Instead, we employ an attentional mechanism during feature aggregation to select the high-frequency features that best match the geodetic features of the gridded data. This mechanism filters the high-frequency noise in the gridded data, achieving an effect similar to that of the VC method. Because we only extract the geodesic features from the gridded data, we also do not need to consider whether the collapsed edges depend on a specific task [27].

**Figure 3:**
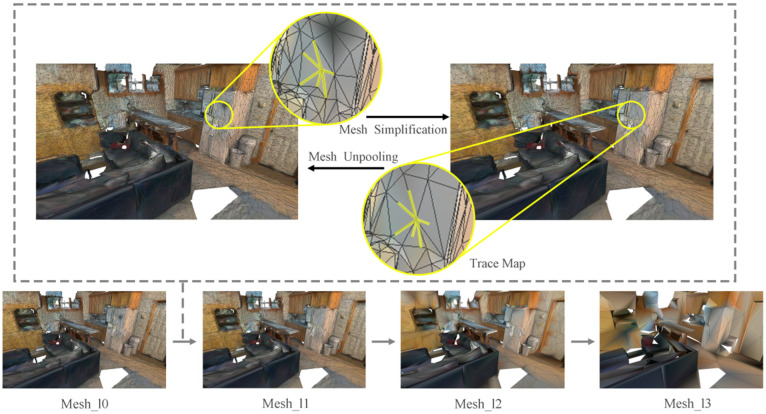
Mesh simplification. Mesh_l0 to Mesh_l3 is part of the mesh simplification process, with the yellow label indicating the trajectory map of a point from Mesh_l0 to Mesh_l1.

Furthermore, the trajectory maps are saved to track pooling between various grid levels. In practice, we set up seven layers to enrich the multi-resolution information, as shown in Figure 3. An effective method of tracking connections between pooled and non-pooled vertices is to use trajectory map, which tracks vertex connectivity to facilitate quick searches between neighboring mesh levels. By improving the handling of multi-resolution hierarchies, we can track these simplified contractions and generate pooled trace maps through pooled trace mapping.

(2) Self-domain Attention: The adjacency matrix $A=[a_{ij}]\in R^{n\times n}$ describes graph G, while the diagonal matrix *D* is defined as $D=diag\left(d_{1},\;d_{2},\ldots ,\;d_{n}\right),\;d_{i}=\sum_{j}a_{ij}$, where $d_{i}$ denotes the degree of vertex i. The formula $D^{-1}A$ represents the graph features of the geodesic branch or the graph features of the Euclidean branch.

To effectively aggregate the vertex features of the mesh, we construct the self-domain attention module in the geodesic branch by referring to reference [28]. The attention weight matrix $a_{ij}$ for each edge from point *j* to point *i* in the mesh data is computed using the following formulas:

(5) $$q_{i}^{\left(l\right)}=W_{q}^{\left(l\right)}D^{-1}A_{geo}^{\left(l\right)}+b_{q}^{\left(l\right)}$$

(6) $$k_{i}^{\left(l\right)}=W_{k}^{\left(l\right)}D^{-1}A_{geo}^{\left(l\right)}+b_{k}^{\left(l\right)}$$

(7) $$\alpha_{ij}^{\left(l\right)}=\frac{\left\langle q_{i}^{\left(l\right)},\;k_{j}^{\left(l\right)}\right\rangle }{\sum_{u\in N\left(i\right)}\left\langle q_{i}^{\left(l\right)},\;k_{u}^{\left(l\right)}\right\rangle }$$

Including $\left\langle q,\;k\right\rangle =\exp\left(\frac{q^{⊤}k}{\sqrt{d}}\right)$, where $q_{i},\;k_{i},\;v_{i}$ correspond to Q, K, V of traditional attention, respectively, and d is the dimension of output. Message aggregation is conducted with the obtained attention weight matrix $\alpha_{ij}$, and the formulas are as follows:

(8) $$v_{j}^{\left(l\right)}=W_{v}^{\left(l\right)}D^{-1}A_{\mathrm{geo}\;}^{\left(l\right)}+b_{v}^{\left(l\right)}$$

(9) k^(l+1)=∑j∈N(i)αij(l)vj(l)

To prevent excessive smoothing of network weights, we add a gated residual connection [29].

The formulas are as follows:

(10) $$r_{i}^{\left(l\right)}=W_{r}^{\left(l\right)}D^{-1}A_{\mathrm{geo}\;}^{\left(l\right)}+b_{r}^{\left(l\right)}$$

(11) $$\beta_{i}^{\left(l\right)}=\mathrm{sigmoid}\left(W_{g}^{\left(l\right)}\left[{\overset{\wedge }{h}}^{\left(l+1\right)};\;r_{i}^{\left(l\right)};\;{\overset{\wedge }{h}}^{\left(l+1\right)}-r_{i}^{\left(l\right)}\right]\right)$$

(12) $$D^{-1}A^{\left(l+1\right)}=\mathrm{Relu}\left(\mathrm{LayerNorm}\left(\left(1-\beta_{i}^{\left(l\right)}\right){\overset{\wedge }{h}}^{\left(l+1\right)}+\beta_{i}^{\left(l\right)}r_{i}^{\left(l\right)}\right)\right)$$

If the self-domain attention module is used in the last output layer, the linear transformation needs to be removed using the following formula:

(13) $$D^{-1}A^{\left(l+1\right)}=\left(1-\beta_{i}^{\left(l\right)}\right){\overset{\wedge }{h}}^{\left(l+1\right)}+\beta_{i}^{\left(l\right)}N_{i}^{\left(l\right)}$$

(3) Cross-domain Attention Module: To fuse mesh data features and features extracted by Euclidean branch, we designed the cross-domain attention module, which mirrors the structure of the self-domain attention module. The difference is the input features. First, when calculating the attention weight matrix $a_{ij}$, we use the Euclidean feature $D^{-1}A_{\mathrm{euc}\;}^{\left(l\right)}$ to calculate $k_{i}^{\left(l\right)}$, identifying Euclidean features that correspond to the current geodesic features. The formula is as follows:

(14) $$k_{i}^{\left(1\right)}=W_{k}^{\left(1\right)}D^{-1}A_{euc}^{\left(l\right)}+b_{r}^{\left(l\right)}$$

Second, when incorporating a gated residual connection, the Euclidean features $D^{-1}A_{\mathrm{euc}\;}^{\left(l\right)}$ are used, represented by the following equation:

(15) $$r_{i}^{\left(l\right)}=W_{r}^{\left(l\right)}D^{-1}A_{\mathrm{euc}\;}^{\left(l\right)}+b_{r}^{\left(l\right)}$$

## 4. Experiment Results and Analyses

### 4.1. Datasets

To demonstrate the validity and robustness of our proposed model, the EGNet, we conducted experiments on two benchmarks: ScanNet v2 and Matterport3D [6,29].

ScanNet v2 [29] updates the annotation of ScanNet, achieving a surface coverage of 90%. This large-scale dataset comprises 2.5 million RGB-D images collected from 1513 scans across 707 different indoor scenes. The dataset’s annotations include camera postures, textured meshes, dense semantic segmentation at the object level, and aligned computer-aided design (CAD) models, which are valuable for scene understanding. ScanNet v2 surpasses previous RGB-D datasets by more than one order of magnitude in size. To evaluate the effectiveness of the EGNet, we performed an ablation study using the ScanNet validation set.

Matterport3D [6] is a new RGB-D dataset that includes 10,800 panoramic views and 194,400 RGB-D images from 90 architectural-scale scenes. This dataset comprehensively provides labels for the walls, floors, ceilings, doors, and windows of each house in a 3D mesh. The dataset is divided into training, verification, and test splits of 61, 11, and 18 scans, respectively. Additionally, we report the average category accuracy scores of 21 categories in the test set.

### 4.2. Implementation Detail

The EGNet was trained and tested on a single 32G GV100GL GPU using Python 3.6 and PyTorch 1.4 in a CUDA10.1 and UBUNTU 18.04 environment. During training, we employed cross-entropy as the loss function for both modules, summing the individual losses to obtain the total loss. We used the stochastic gradient descent (SGD) optimizer with a poly scheduler to minimize the loss, in which the initial learning rate was initially set to 0.1 and the power to 0.9. The maximum number of iterations was set to 80,000; once the iteration reached this limit, the learning rate decayed to its final value. Considering the hardware limitations, the batch size was set to six.

To improve the robustness of the model, we trained the network without cropping the dataset using a seven-level mesh simplification process. This included random edge dropping, color dithering, and random scaling at each level of the mesh. Specifically, we applied VC on the input grid at levels 1 and 2 based on a unit length of two cm, while the QEM was used for the remaining five levels to reduce the number of vertices to reach the 30% simplification target. The final data input to the network was organized as a dictionary containing eight levels of vertices, seven levels of trajectories, raw colors, labels, and additional features.

To evaluate the performance of the EGNet in the task of semantic segmentation, we validated the effectiveness of our proposed method using both the Scannetv2 and Matterport3D datasets. On the Scannetv2 dataset, we adopted two commonly used evaluation metrics: the mean average precision (mAP) and mean class intersection over union (mIoU). On the Matterport3D dataset, we employed the mAcc metric, which reports the mAcc for 20 categories. The mAP measures the detection accuracy and coverage of the model across different semantic categories, combining both precision and recall. The mIoU evaluates the average overlap between the predicted regions and the ground-truth regions for each semantic category. The mAcc assesses the classification accuracy of the prediction results compared to the true results at each pixel point.

### 4.3. Experimental Results and Analysis

**Figure 4:**
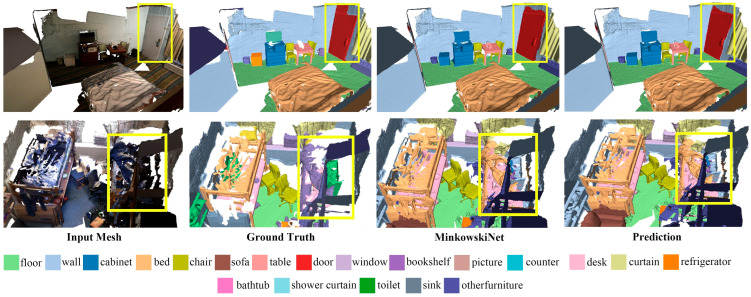
Results of the ScanNet v2 validation. We have highlighted the main differences with yellow bounding boxes. Observing the segmentation results of the door in the first instance, our method demonstrates more accurate boundary segmentation. In the second instance, despite the poor quality of the environmental scan, although the MinkowskiNet method also identified the unannotated bed, a closer inspection reveals that our method provides a clearer segmentation boundary between the bed and the desk.

The performance of the proposed network for indoor scene segmentation was evaluated through experiments conducted on the ScanNet V2 dataset; Table 1 presents the results. Our method achieved a mean intersection over union (mIoU) of 73.3% on the validation set and 74.1% on the test set. Compared with DCM-Net, the mIoU performance improved by 8.3%; compared with the leading sparse convolution method (i.e., SparseConvNet), the mIoU increased by 1.6%. The proposed method outperformed RFCR+KPConv on the test set by 3.8%, as it employs a cross-modal feature complementation strategy, directly fuses features at the semantic level, avoids over-reliance on local features, and effectively models complex geometric and topological structures. While the AF-GCN method optimizes the traditional UNet architecture by combining graph convolutional networks (GCNs) and geometric attention modules (GAFs), it has certain disadvantages in computational complexity, model complexity, and handling large-scale data. Although our method’s performance on the validation set is 0.1% lower, it outperforms AF-GCN by 2.3% on the test set, fully demonstrating the superiority of our method in terms of computational efficiency and global geometric structure modeling. Although the PointTransformer series achieved better results on the ScanNet dataset, its neglect of the geodesic information on object surfaces may limit its performance in tasks requiring a high boundary segmentation accuracy. For example, in robotic arm-grasping path-planning tasks, the boundary is often used to determine the homogeneous transformation matrix of the gripper for a grasp pose. As shown in Table 2, the proposed method demonstrated superior mAP performance, indicating its capability to meet the requirements for a high boundary segmentation accuracy in tasks such as the spatial positioning of operational targets for indoor wheeled robots. Figure 4 shows the results of our model on the ScanNet V2 dataset. Additionally, we compared it with other methods, demonstrating its effectiveness in predicting challenging categories, such as storage cabinets and curtains, and in edge segmentation accuracy.

**Figure 5:**
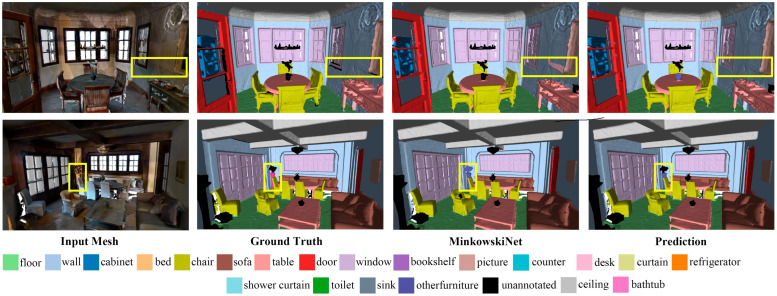
Visualization of Matterport3D. We have highlighted the main differences with yellow bounding boxes. In the first example, our method shows almost no errors compared to the ground-truth labels and achieves more accurate segmentation regions than other methods. Additionally, our method successfully identifies the flowerpot (other furniture) on the table. In the second example, despite errors in the ground-truth labels, our method achieves more accurate target classification compared to other methods.

Additionally, Table 3 presents the overall evaluation results of the EGNet on the Matterport3D benchmark, showing the mean accuracy (mAcc) across 20 categories. Our model’s overall mean accuracy (omAcc) is 0.3% higher than that of the previous state-of-the-art methods. Although the improvement is modest, our method effectively produced accurate predictions even in cases in which the original annotations might have been incorrect or missing. Figure 5 shows a comparison with other methods. Our method demonstrates a superior accuracy and edge segmentation precision compared to the MinkowskiNet method on the Matterport3D dataset, and it can correctly identify targets even with missing or erroneous annotations.

**Figure 6:**
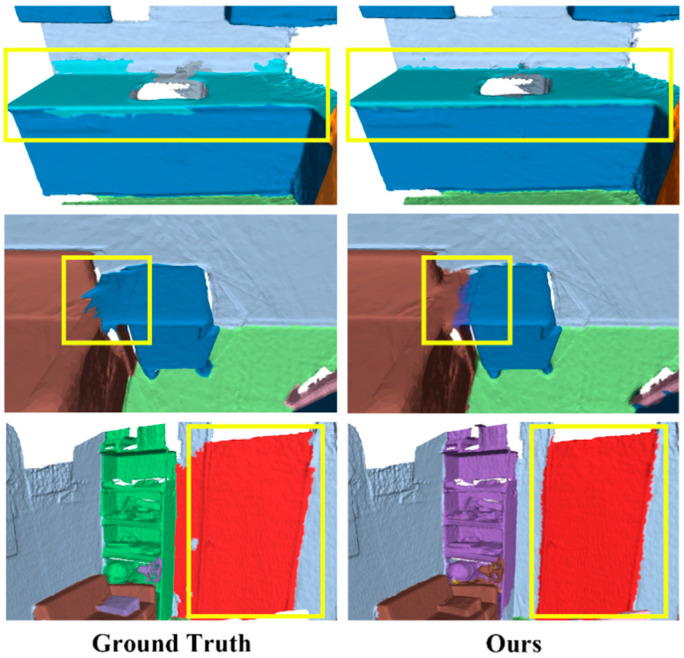
Detailed local regions from the ScanNetV2 validation set, with key differences marked by yellow bounding boxes. The first example focuses on a kitchen corner, where EGNet produces smoother segmentation results compared to the ground-truth annotations. The second and third examples depict bedroom scenes, for which our method successfully distinguishes cabinets from sofas even in areas with blurred boundaries. Notably, in the third example, there was a significant improvement in door recognition, and our method correctly classifies a shelf that had been mislabeled as part of the floor.

In robotic arm-grasping path planning, accurately identifying target boundaries is crucial for determining the homogeneous transformation matrix of the gripper, thereby ensuring precise grasping poses. Figure 6 shows a detailed visualization of local features in the ScanNet validation set, with key differences highlighted in yellow bounding boxes. The first example focuses on a kitchen corner, for which the EGNet demonstrates smoother and more refined segmentation results compared to the ground-truth annotations. The second and third examples shift to bedroom scenes. Even in cases of blurred boundaries, our method accurately distinguishes cabinets from sofas. Notably, the third example reveals a significant improvement in recognizing the door, in which discrepancies are evident. Furthermore, our method correctly identifies a shelf misannotated as part of the floor, treating it as a distinct object. These findings hold significant practical value for grasping tasks, as clear segmentation boundaries are critical for improving the accuracy of target boundary localization in robotic arm operations.

### 4.4. Ablation Study

In this section, we conduct ablation experiments on the Scannet V2 for network components to further highlight the significance of building modules in the EGNet. In all the experiments, we ensured consistent parameters throughout the study.

To validate our approach, we conducted an ablation study based on the preliminary assessment of combining the data from the two modules. We compared the EGNet to two baseline networks: “Euc Only”, a U-Net structure based on a sparse convolution operating on voxels, and “Geo Only”, a network with an identical structure based on a graph convolution of multi-level mesh simplification information.

Table 4 demonstrates that using the two branches in parallel significantly enhanced the network’s segmentation performance. In our experiments, both branches were implemented using the U-Net structure. The difference between two branches was the number of parameters. To mitigate the difference, we increased the number of channels and layers in the geodesic branch to obtain an approximately 1.5-times-narrower gap. Ultimately, the two modules showed comparable overall performances. The “Geo only” module outperformed the “Euc only” module by 1.3% in mIoU. By combining the two branches, we achieved an mIoU of 73.3%, an mAcc of 80.4%, and an OA of 90.6%.

**Figure 7:**
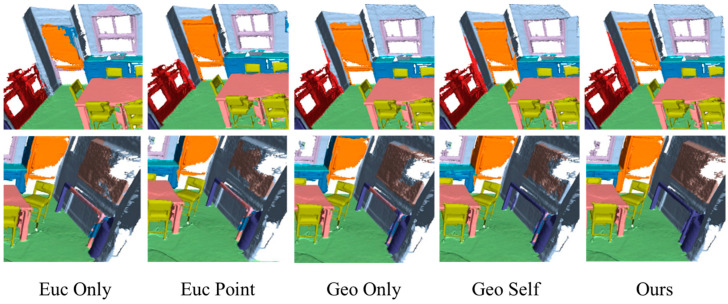
Visualization results of the ablation study. The data in the third column show that the accuracy of edge segmentation is significantly improved with the introduction of geodesic branching. The effectiveness of our proposed cross-domain attention module is demonstrated by comparing the results of the data in the fourth and fifth columns.

Additionally, we compared the proposed model with other modules. The “Euc Point” module extended the “Euc Only” module with a point-based branch by transforming and combining the information contained in voxels and points. It compensated for the voxelization-related information error and allowed the model to concentrate on the intricate details of the interior space. As shown in Figure 7, by integrating sparse vertex features from the Euclidean branch through our proposed cross-domain attention module, we have addressed the issue in which the gradients generated during the backpropagation process are not sufficient to effectively reduce the wrong weights’ (i.e., the deviation of the probability distribution) output by the Euclidean branch, which was caused by solely using the method of weight matrices to fuse the mesh data features extracted by the self-domain attention module with those of the Euclidean branch (as illustrated in the fourth column of Figure 7). This has resulted in smoother and more accurate edge segmentation. Furthermore, the introduction of original mesh vertices has reduced the classification error rate caused by extracting Euclidean features solely from voxels, as the probability distribution of points in the devoxelization process has been optimized during the backpropagation. The results from Table 3 highlight the effectiveness of this improvement, resulting in an improvement of 1.8% in the mIoU, 1.4% in the mAcc, and 0.4% in the OA compared with the “Euc Only” module. The “Geo Self” module is an enhancement of the “Geo Only” module, utilizing a self-attention mechanism to effectively aggregate geodesic features from the mesh and improve local information. The “Geo Self” module achieved an mIoU of 70.9%, mAcc of 79.3%, and OA of 89.4%, as shown in Table 3. While its performance was only slightly superior to the “Geo Only” module, a 2.4% improvement was achieved through the fusion block. This block integrated information from the Euclidean module into the geodesic module, enabling the adaptive fusion of features from the two domains.

### 4.5. Model Efficiency

Our proposed model has a parameter count of 44.3 million when the batch size is set to one during the training phase, with a latency of 326 ms and a memory usage of 4.7 GB. During the inference phase, the latency is reduced to 49 ms with 1.5 GB of memory. When using the NVIDIA ORIN edge computing device, the latency is 300 ms. The device is equipped with 512 tensor computation units based on the Volta architecture, an eight-core ARM-based CPU, and 32 GB of memory. This configuration efficiently supports tasks such as scene comprehension, target edge detection, and robotic arm-grasping path planning.

## 5. Conclusions

This study proposes a semantic segmentation network named the EGNet for indoor scenes with mesh data as input. It contains Euclidean and geodesic branches. The Euclidean branch extracts detailed features and voxelized contour features from point cloud data, while the geodesic branch extracts geodesic features from mesh data by graph message propagation. The features of point, voxel, and mesh data are fused by self-attention and cross-domain attention modules. The Matterport3D and ScanNet v2 datasets are used to demonstrate the effectiveness of the EGNet. The results indicate that the fusion is effective for semantic segmentation. This study informs the research on the potential of integrating both Euclidean and geodesic features in semantic segmentation. The EGNet has broad application prospects, especially in robotic arm-grasping path planning tasks. These tasks typically require the precise boundary identification of the target to determine the homogeneous transformation matrix of the gripper, ensuring an accurate grasping pose. Given the EGNet’s outstanding performance in boundary segmentation accuracy, it can meet the high-precision requirements for the spatial localization of operational targets in indoor environments.

## Figures and Tables

**Table 1:** Results of semantic segmentation in Scannet V2 dataset.

Method	Year	Val	Test
PointNet++ [7]	2017	53.5	55.7
3DMV [30]	2018	-	48.4
PointCNN [31]	2018	-	45.8
SparseConvNet [20]	2018	69.3	72.5
MVPNet [32]	2019	-	64.1
PointConv [33]	2019	61.0	66.6
KPConv [34]	2019	69.2	68.6
MinkowskiNet [22]	2019	72.2	73.6
SPH3D-GCN [35]	2019	-	61.8
DCM-Net [18]	2020	-	65.8
RFCR+KPConv [36]	2021	-	70.3
PointTransformer V2 [11]	2022	75.4	74.2
AF-GCN [37]	2023	73.4	71.8
GroupContrast [38]	2024	75.7	
PointTransformer V3 [39]	2024	77.5	77.9
Ours	2024	73.3	74.1

**Table 2:** Results of instance segmentation in Scannet V2 dataset.

Method	${mAP}_{25}$	${mAP}_{50}$	mAP
MinkowskiNet [22]	72.8	56.9	36.0
PointTransformer V2 [11]	76.3	60.0	38.3
Ours	76.1	60.7	40.2

**Table 3:** Matterport3D test category results.

Method	PointNet++ [7]	SplatNet [40]	ScanComplete [41]	TangentConv [42]	3DMV [30]	DCM-Net [18]	VMNet [19]	Ours
**omAcc**	43.8	26.7	44.9	46.8	56.1	66.2	67.2	**67.5**
**Wall**	80.1	90.8	79.0	56.0	79.6	78.4	85.9	84.6
**Floor**	81.3	95.7	95.9	87.7	95.5	93.6	94.4	93.8
**Cab**	34.1	30.3	31.9	41.5	59.7	64.5	56.2	56.1
**Bed**	71.8	19.9	70.4	73.6	82.3	89.5	89.5	89.5
**Chair**	59.7	77.6	68.7	60.7	70.5	70.0	83.7	83.5
**Sofa**	63.5	36.9	41.4	69.3	73.3	85.3	70.0	69.7
**Table**	58.1	19.8	35.1	38.1	48.5	46.1	54.0	53.2
**Door**	49.6	33.6	32.0	55.0	64.3	81.3	76.7	75.4
**Wind**	28.7	15.8	37.5	30.7	55.7	63.4	63.2	63.1
**Bookshelf**	1.1	15.7	17.5	33.9	8.3	43.7	44.6	45.0
**Image**	34.3	0	27.0	50.6	55.4	73.2	72.1	72
**Counter**	10.1	0	37.2	38.4	34.8	39.9	29.1	30.2
**Desk**	0	0	11.8	19.7	2.4	47.9	38.4	48.3
**Window**	68.8	12.3	50.4	48.0	80.1	60.3	79.7	67.7
**Ceiling**	79.3	75.7	97.6	45.1	94.8	89.3	94.5	82
**Refrigerator**	0	0	0.1	22.6	4.7	65.8	47.6	64.7
**Bathtub**	29.0	0	15.7	35.9	54.0	43.7	80.1	83.5
**Toilet**	70.4	10.4	74.9	50.7	71.1	86.0	85.0	77.5
**Sink**	29.4	4.1	44.4	49.3	47.5	49.6	49.2	51.0
**Shower**	62.1	20.3	53.5	56.4	76.7	87.5	88.0	83.4
**Other Furniture**	8.5	1.7	21.8	16.6	19.9	31.1	29.0	43.4

The bold ones are the best results, and the underlined ones are the second-best results.

**Table 4:** Ablation study for models with different inputs.

	Model Part			Result		
	Point	Self-Domain	Cross-Domain	mIou	mAcc	OA
Euc Only				68.3	77.2	88.6
Euc Point	✓			70.1	78.6	89
Geo Only				69.6	77.9	88.9
Geo Self		✓		70.9	79.3	89.4
Ours	✓	✓	✓	73.3	80.4	90.6

## Data Availability

Our code is available at: https://github.com/ritajin6/pvm-net/tree/main, accessed on 19 December 2024.
